# Identification of Two Long Non-Coding RNAs AC010082.1 and AC011443.1 as Biomarkers of Coronary Heart Disease Based on Logistic Stepwise Regression Prediction Model

**DOI:** 10.3389/fgene.2021.780431

**Published:** 2021-11-18

**Authors:** Chao Liu, Lanchun Liu, Jialiang Gao, Jie Wang, Yongmei Liu

**Affiliations:** ^1^ Guang’anmen Hospital, China Academy of Chinese Medical Sciences, Beijing, China; ^2^ Graduate School, Beijing University of Chinese Medicine, Beijing, China; ^3^ Key Technology Laboratory of Cardiovascular Disease-Syndrome Combination, Guang’anmen Hospital, China Academy of Chinese Medical Sciences, Beijing, China

**Keywords:** long non-coding RNAs, biomarkers, competing endogenous RNA, logistic stepwise regression, RT-qPCR

## Abstract

Coronary heart disease (CHD) is a global health concern with high morbidity and mortality rates. This study aimed to identify the possible long non-coding RNA (lncRNA) biomarkers of CHD. The lncRNA- and mRNA-related data of patients with CHD were downloaded from the Gene Expression Omnibus database (GSE113079). The limma package was used to identify differentially expressed lncRNAs and mRNAs (DElncRNAs and DEmRNAs, respectively). Then, miRcode, TargetScan, miRDB, and miRTarBase databases were used to form the competing endogenous RNA (ceRNA) network. Furthermore, SPSS Modeler 18.0 was used to construct a logistic stepwise regression prediction model for CHD diagnosis based on DElncRNAs. Of the microarray data, 70% was used as a training set and 30% as a test set. Moreover, a validation cohort including 30 patients with CHD and 30 healthy controls was used to verify the hub lncRNA expression through real-time reverse transcription-quantitative PCR (RT-qPCR). A total of 185 DElncRNAs (114 upregulated and 71 downregulated) and 382 DEmRNAs (162 upregulated and 220 downregulated) between CHD and healthy controls were identified from the microarray data. Furthermore, through bioinformatics prediction, a 38 lncRNA-21miRNA-40 mRNA ceRNA network was constructed. Next, by constructing a logistic stepwise regression prediction model for 38 DElncRNAs, we screened two hub lncRNAs AC010082.1 and AC011443.1 (*p* < 0.05). The sensitivity, specificity, and area under the curve were 98.41%, 100%, and 0.995, respectively, for the training set and 93.33%, 91.67%, and 0.983, respectively, for the test set. We further verified the significant upregulation of AC010082.1 (*p* < 0.01) and AC011443.1 (*p* < 0.05) in patients with CHD using RT-qPCR in the validation cohort. Our results suggest that lncRNA AC010082.1 and AC011443.1 are potential biomarkers of CHD. Their pathological mechanism in CHD requires further validation.

## Introduction

Globally, coronary heart disease (CHD) is a major disease with high morbidity and mortality, which seriously threatens human health (Collet et al., 2021), killing approximately 8 million people annually worldwide (Mortality and Causes of Death, 2015). Reportedly, > 700,000 people die from CHD annually in China, and the overall mortality rate has been increasing each year (Wang et al., 2011). CHD is a complex multifactorial disease with many risk factors, such as age, hypertension, diabetes, dyslipidemia, and smoking status (Knuuti et al., 2020). The definitive diagnosis of CHD primarily depends on invasive coronary angiography, which is relatively costly, time-consuming, and uncomfortable for the patient (Novak et al., 2021). However, CHD in its early stage is not easily detected by routine examinations, such as electrocardiography and cardiac ultrasound, resulting in a high mortality rate of CHD (Miao et al., 2019). Therefore, it is necessary to identify a novel non-invasive biomarker in the early stages of CHD, to enable early diagnosis and prevention of CHD.

Evidence in recent years has showed that long non-coding RNAs (lncRNAs) have a key role in the gene regulation and widely concerned (Statello et al., 2020; Zhang et al., 2020; Hongkuan et al., 2021). LncRNAs are non-coding RNAs with a transcription length >200 bp and lack protein-coding potential. A variety of cellular functions within the nucleus and cytoplasm could be regulated by lncRNAs, which are important for normal development and disease progression (Lu and Thum, 2019). Due to their tissue-specific and condition-specific expression patterns, lncRNAs can be used as biomarkers and therapeutic targets for multiple diseases in blood, plasma, and urine (Beermann et al., 2016). A variety of lncRNAs, such as lncRNA OTTHUMT00000387022 (Cai et al., 2016), AC100865.1 (Yang et al., 2015), and uc010yfd.1 (Li et al., 2018), are abnormally expressed in CHD and therefore, can be used as potential biomarkers of CHD. LncRNAs play key roles in specific physiological and pathological processes of CHD, including the induction of vascular smooth muscle cell proliferation, apoptosis, lipid metabolism, and inflammation (Li et al., 2020; Li et al., 2021a). LncRNAs have been considered promising regulatory genes or biomarkers for CHD (Lu and Thum, 2019).

Recently, the proposed competing endogenous RNAs (ceRNAs) hypothesis has provided the possibility for further study of molecular mechanisms underlying several diseased conditions (Cheng et al., 2015; Ouyang et al., 2020; Wang et al., 2020). CeRNAs can act as miRNA sponges and regulate mRNA expression through their miRNA response elements (MREs) (Salmena et al., 2011). A number of studies have shown that lncRNA can regulate the progression of CHD through the ceRNA mechanism. For instance, lncRNA HCG11 affects the expression of FOXF1 by targeting miR-144-3p, thereby regulating the proliferation and apoptosis of vascular smooth muscle cells in atherosclerosis (Liu et al., 2020). The lncRNA HOTAIR can protect myocardial infraction and hypoxia-induced cardiomyocyte apoptosis by interacting with miR-519d-3p (Zhang et al., 2019). In the present study, we aimed to construct the ceRNA regulatory network of CHD using the Gene Expression Omnibus (GEO) database and screen the possible lncRNAs as biomarkers of CHD.

## Materials and Methods

### Data Acquisition and Preprocessing

The gene expression profile of human CHD was obtained from the GEO (https://www.ncbi.nlm.nih.gov/geo) with accession number GSE113079. This dataset included 93 patients with CHD and 48 healthy controls using the GPL20115 platform (Agilent-067406 Human CBC lncRNA + mRNA microarray V4.0). LncRNA and mRNA expression data from the GPL20115 platform were obtained by reannotating microarray probes according to the probe set sequences and the annotations of protein-coding genes and lncRNA records in GENCODE (GRCh38, release 35, http://www.gencodegenes.org/). The BLASTn (https://ftp.ncbi.nlm.nih.gov/blast/executables/LATEST/) was used to align the probe sequences with those of non-coding and coding transcript sequences from GENCODE. The transformed data (lincRNA, antisense, sense_overlapping, processed_transcript, 3prime_overlapping_ncrna, ncrna_host, bidirectional_promoter_lncrna, ambiguous_orf, retained_intron and sense_intronic) were considered as lncRNAs.

### Screening Differentially Expressed lncRNAs (DElncRNAs) and mRNAs (DEmRNAs)

The identification of DElncRNAs and DEmRNAs between patients with CHD and healthy controls were performed by using the “limma” package of R software (version 4.0.1) (R Core Team, 2018). An independent two-sample *t*-test and the false discovery rate (FDR) adjusted using the Benjamini–Hochberg method were used to analyze the DElncRNAs and DEmRNAs (Long et al., 2019). *p* < 0.05, FDR <0.05, and |log_2_ fold change (FC)| > 1 were considered as the cut-off criteria.

### Target Genes Prediction and Construction of the ceRNA Network

**FIGURE 1:**
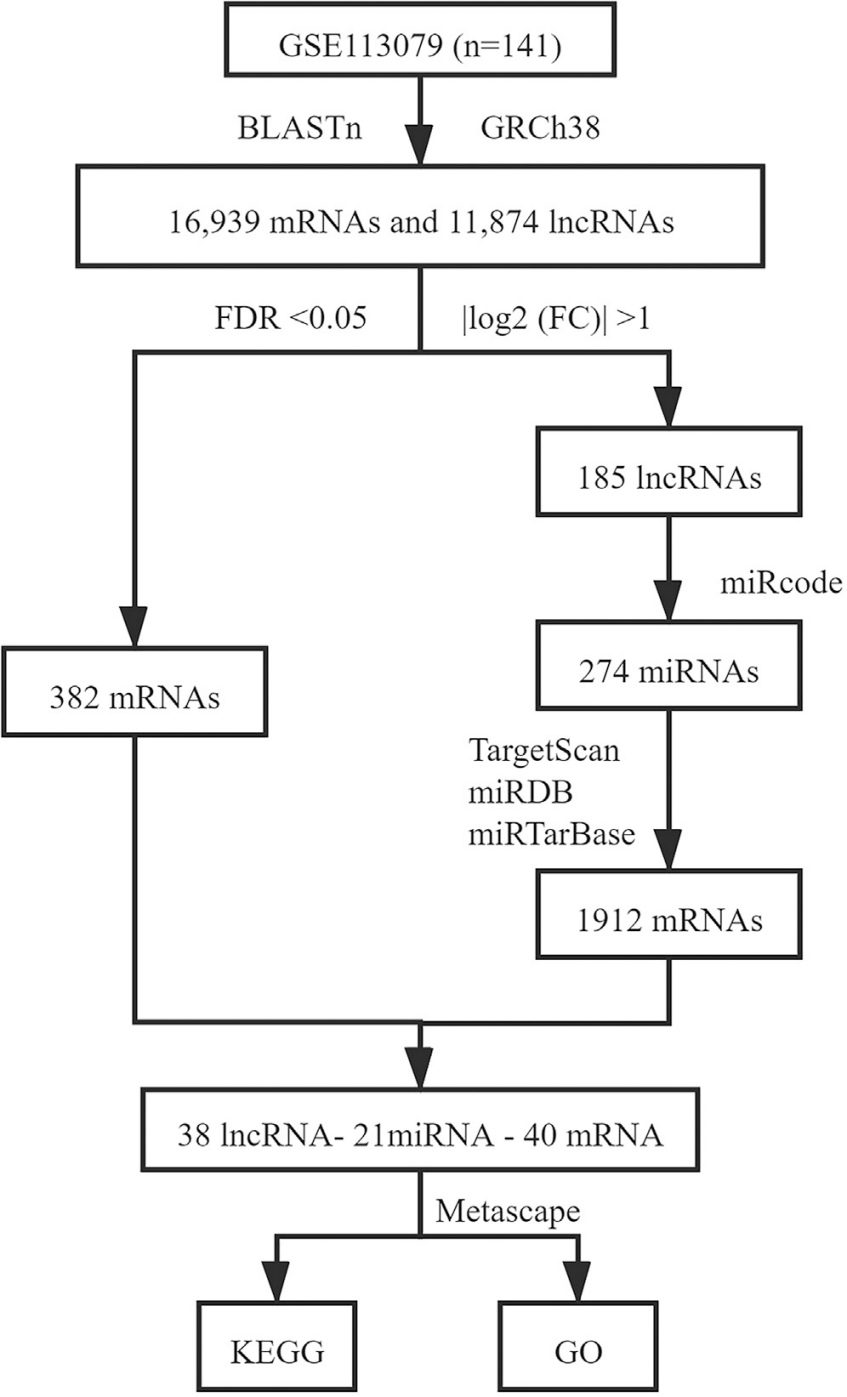
ceRNA network construction process.

The prediction of lncRNAs to target miRNAs was conducted using the miRcode database (Jeggari et al., 2012). DEmRNAs targeted by differentially expressed miRNAs (DEmiRNAs) were retrieved based on the TargetScan, miRDB, and mirtarbase databases, and only those recognized by these three databases were considered candidate DEmRNAs (Agarwal et al., 2015; Wong and Wang, 2015; Chou et al., 2016). Next, the predicted target DEmRNAs and previous identified DEmRNAs were intersected. Based on the above lncRNA-miRNA and miRNA-mRNA interactions, the lncRNA-miRNA-mRNA network was visualized using Cytoscape 3.8.0 software (Shannon et al., 2003). The R package ‘‘pheatmap’’ was used to draw heatmaps for DElncRNAs and DEmRNAs. Figure 1 shows the process used for ceRNA network construction.

### Functional and Pathway Enrichment Analysis of DEmRNAs

To clarify the potential biological processes of DEmRNAs associated with the ceRNA network, we used Metascape (http://metascape.org) to analyze the Gene Ontology (GO) and Kyoto Encyclopedia of Genes and Genomes (KEGG) functional enrichment of the DEmRNAs (Zhou et al., 2019). *p* < 0.05, min overlap mRNAs≥2, and enrichment factor >1.5 were considered to be statistically significant (Gao et al., 2020).

### Constructing a Logistic Stepwise Regression Prediction Model and Screening Hub lncRNAs

**FIGURE 2:**
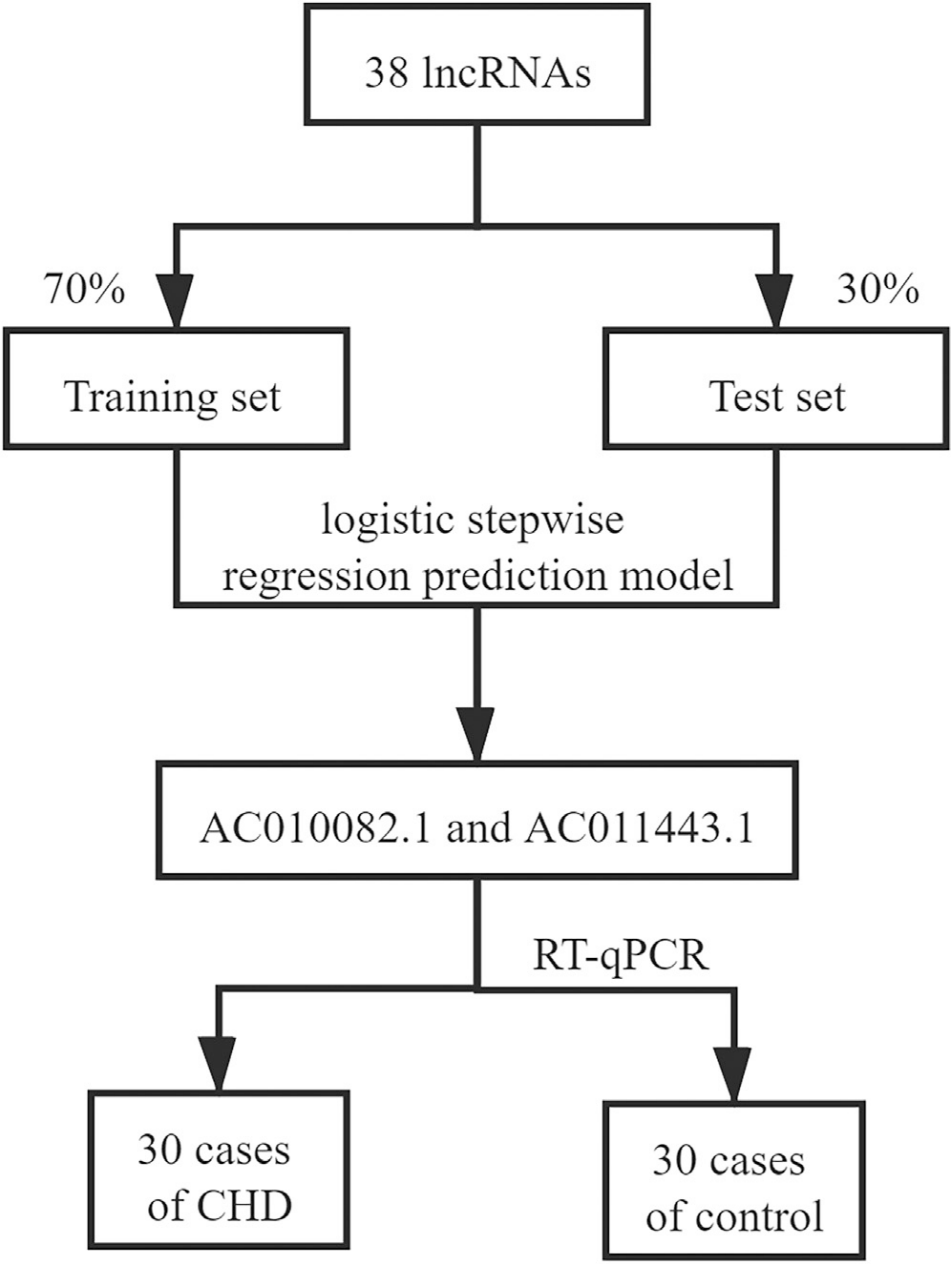
Screening and validating hub lncRNAs.

The 141 samples from the GSE113079 dataset were randomly divided into training (70% of the samples, 63 patients with CHD and 36 healthy controls) and test (30%, 30 patients with CHD and 12 healthy controls) sets, and they maintained the similar ratio for patients with CHD and healthy controls (*p* = 0.372). SPSS Modeler 18.0 (IBM Canada Ltd., Markham, Ontario, Canada) was used to construct a logistic stepwise regression prediction model for CHD diagnosis based on DElncRNAs. Further, the sensitivity and specificity of the model were calculated using SPSS Modeler. The receiver operating characteristic (ROC) curve was used to evaluate the effectiveness of the classification model, and the area under the curve (AUC) value was calculated from the ROC curve. ROC curves were calculated using the R package “pROC.” Principal component analysis (PCA) using the R package “ggplot2” was performed on the screened hub lncRNAs, and their expression levels in patients with CHD and healthy controls were analyzed. The selection process for screening hub lncRNAs is shown in Figure 2.

### Study Population

A total of 30 patients with CHD (17 males) and 30 healthy controls (6 males) were recruited in Guang’anmen Hospital, Beijing, China, as the validation cohort. This study complied with the Declaration of Helsinki and was approved by the Ethics Committee of Guang’anmen Hospital, China Academy of Chinese Medical Sciences (no. 2019–224-KY). All subjects were aged between 42 and 77 years. CHD diagnosis was based on the European Society of Cardiology criteria, which specifies at least one vessel lesion (>50% narrowing of luminal diameter) using coronary angiography (Task Force et al., 2013). Healthy controls without chronic disease or acute infection in the last 2 weeks were recruited from the medical examination center of Guang’anmen Hospital. All participants signed an informed consent upon receiving a complete explanation of the study.

### Total RNA Isolation and Reverse Transcription

Peripheral blood samples (4–8 ml) were collected in the early morning from patients with CHD and healthy controls using EDTA vacuum anticoagulant blood vessels. Peripheral blood mononuclear cells (PBMCs) were isolated using the Red Blood Cell Lysis kit (TIANGEN, Beijing, China) and total RNA was extracted from PBMCs using TRIzol reagent (Invitrogen, Carlsbad, CA, United States) according to the manufacturers’ instructions. RNA quality and quantity were evaluated using a NanoDrop 2000 spectrophotometer (Thermo Fisher Scientific, MA, United States). LncRNA reverse transcription was performed using the lncRcute lncRNA First-Strand cDNA Kit (TIANGEN) from 1 µg total RNA according to the manufacturer’s instructions.

### Validation of Hub lncRNAs Using RT-qPCR

Reverse transcription-quantitative PCR (RT-qPCR) was performed using the lncRcute lncRNA qPCR kit (TIANGEN) to detect relative expression using the standard protocols on the ABI7900HT Fast Real-Time PCR system (Applied Biosystems, MA, United States). *ACTB* was used as an internal reference. The cycling PCR conditions were: 95°C for 3 min; 40 cycles of 95°C for 5 s, 55°C for 10 s, and 72°C for 15 s; 95°C for 15 s, 65°C for 15 s, and 95°C for 15 s. The primer sequences are as follows: AC010082.1 forward primer, 5′-TTT​GGT​CTA​GGC​GCT​AGG​AAT-3′; AC010082.1 reverse primer, 5′-CTT​TTC​CCC​TTA​CCC​TGC​TTT-3′; AC011443.1 forward primer, 5′-TGT​TCC​AAG​GTC​AAC​CAA​AAA-3′; AC011443.1 reverse primer, 5′-CCA​AGG​TGG​TCA​AAT​CTG​TGT-3′; *ß*-actin forward primer, 5′-GAG​ACC​TTC​AAC​ACC​CCA​GCC-3′; *ß*-actin reverse primer, 5′-AAT​GTC​ACG​CAC​GAT​TTC​CC-3′. Relative expression data were analyzed using the 2^-∆∆CT^ method (Livak and Schmittgen, 2001). R 4.0.1 software was used to analyze differences in expression.

## Results

### DElncRNAs and DEmRNAs

**FIGURE 3:**
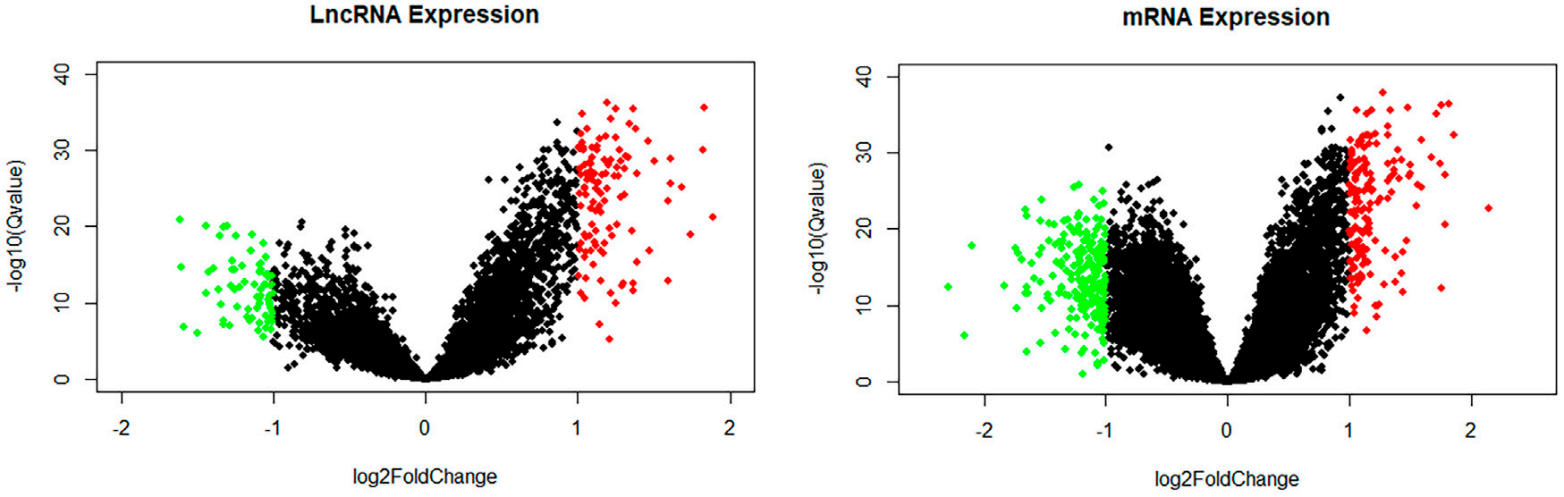
Volcano map of lncRNAs and mRNAs. Green spots represent downregulated DElncRNAs or DEmRNAs; red spots represent upregulated DElncRNAs or DEmRNAs.

In all, 16,939 mRNAs and 11,874 lncRNAs were derived from the microarray data by conducting the GENCODE probe reannotation. Using *p* < 0.05, FDR <0.05, and |log2(FC)| > 1 as screening criteria, 185 DElncRNAs (114 upregulated and 71 downregulated) and 382 DEmRNAs (162 upregulated and 220 downregulated) between CHD and healthy controls were identified. The volcano maps of DElncRNAs and DEmRNAs are shown in Figure 3.

### Construction of the ceRNA Network

**FIGURE 4:**
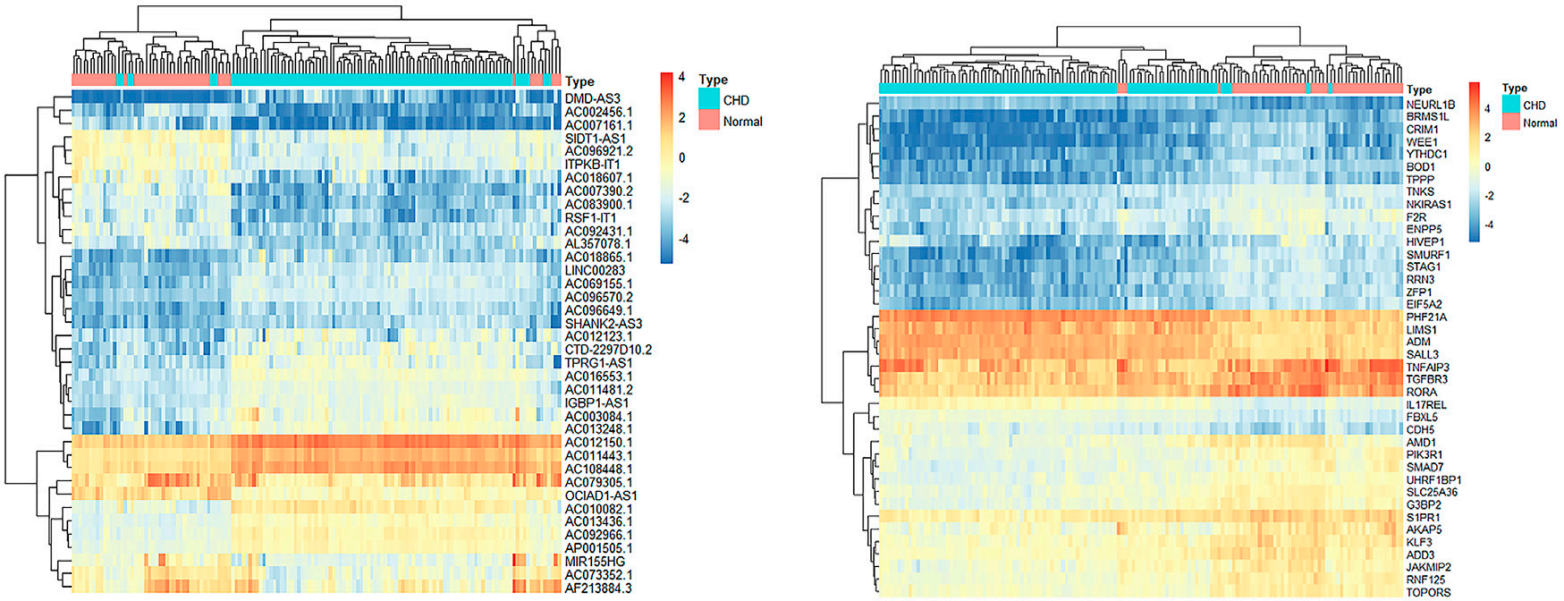
Heatmaps of DElncRNAs and DEmRNAs.

**FIGURE 5:**
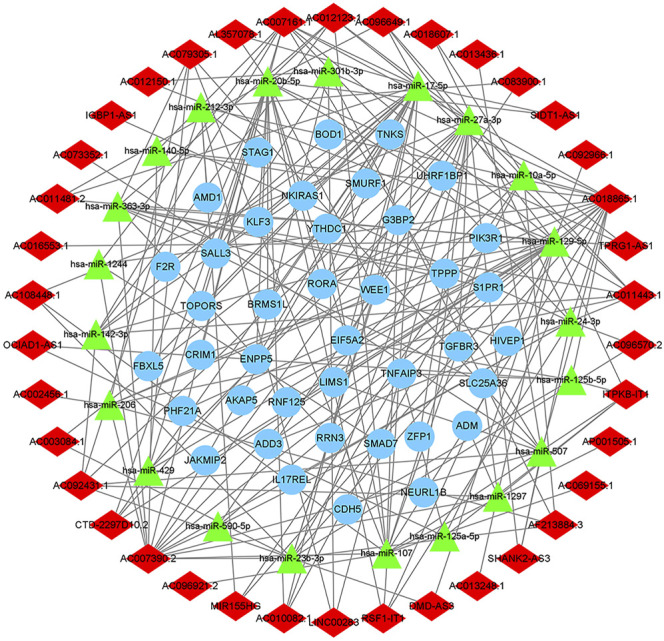
The ceRNA network of lncRNA-miRNA-mRNA. The light blue round nodes, red diamond nodes, and green triangle nodes indicate mRNA, lncRNA, and miRNA, respectively.

We used the miRcode database to predict miRNA-targeted lncRNAs. The constructed ceRNA network only included interactions between the DEmiRNAs and DElncRNAs; thus, 1794 interactions between 47 DElncRNAs (including AC011443.1, AC010082.1, and LIN00283) and 274 DEmiRNAs (including hsa-mir-27a-3p, hsa-mir-206, and hsa-mir-1244) were identified. We mapped the abovementioned 274 DEmiRNAs against TargetScan, miRDB, and miRTarBase to search for target DEmRNAs. A total of 1912 DEmRNAs that interacted with 40 of the 274 DEmiRNAs in all three datasets were selected. After intersecting the predicted 1912 DEmRNAs and previous 382 DEmRNAs, a 38 lncRNA-21 miRNA-40 mRNA ceRNA network was constructed (Figures 4, 5).

### Functional Enrichment Analysis

**FIGURE 6:**
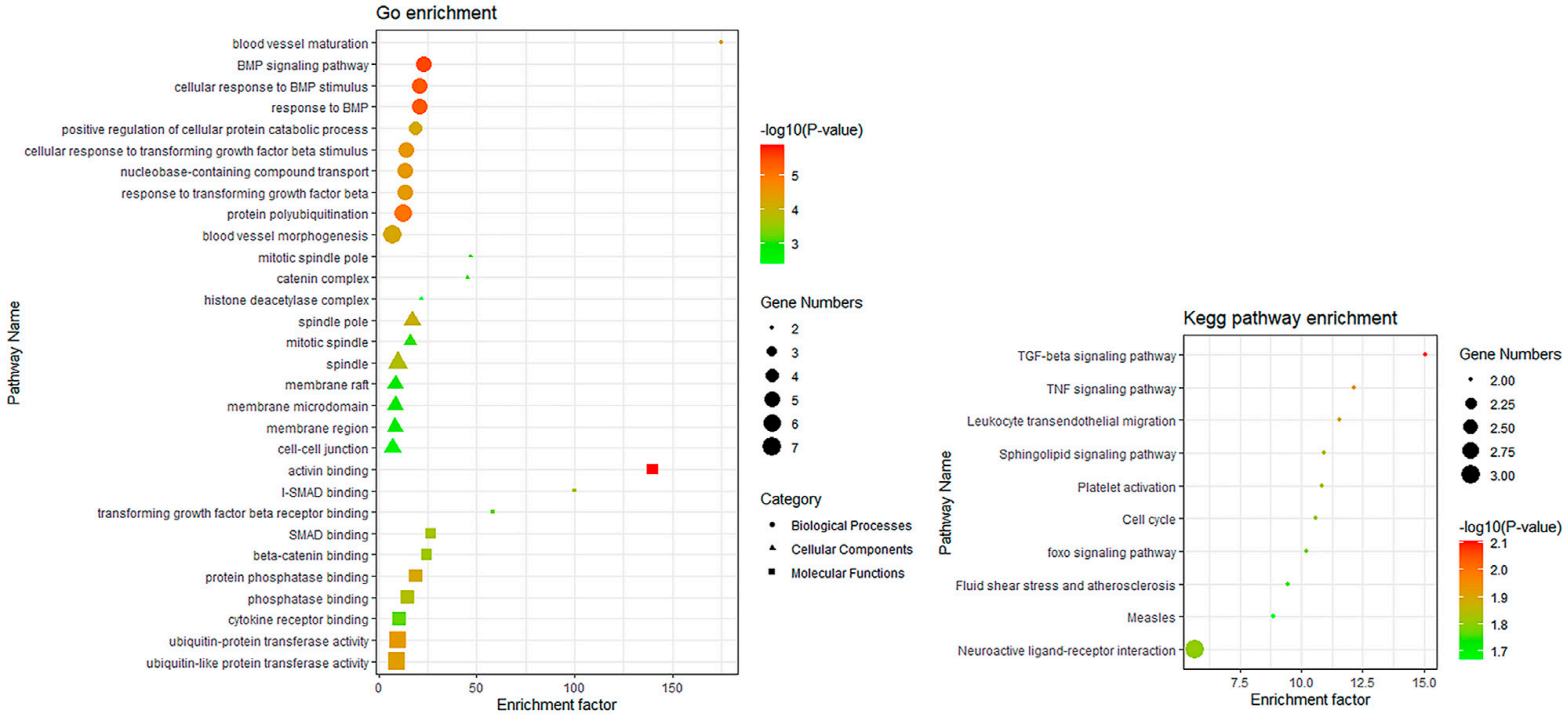
Top 10 terms obtained using GO functional and KEGG pathway enrichment analyses.

To determine the biological functions and pathways of the 40 DEmRNAs in the constructed ceRNA network, we used the Metascape database to analyze the GO functional enrichment and KEGG pathway enrichment (Figure 6). The top terms of biological processes were “BMP signaling pathway,” “protein polyubiquitination,” and “cellular response to transforming growth factor beta stimulus”; the top cellular component terms were “spindle pole,” “mitotic spindle pole,” and “catenin complex”; the top molecular function terms were “activin binding,” “ubiquitin-protein transferase activity,” and “transforming growth factor beta receptor binding.” According to KEGG pathway analyses, the most significant pathways included “TGF-beta signaling pathway,” “TNF signaling pathway,” and “leukocyte transendothelial migration.”

### Logistic Stepwise Regression Prediction Model Based on DElncRNAs

By setting 38 DElncRNAs as independent variables, we constructed a logistic stepwise regression prediction model for CHD. The analysis reveals that AC010082.1 and AC011443.1 (*p* < 0.05) as lncRNAs were statistically significant for model construction among the 38 independent variables. The prediction model formula is:

Logit(P) = −13.718 + AC010082.1*8.099 + AC011443.1*3.825.

**FIGURE 7:**
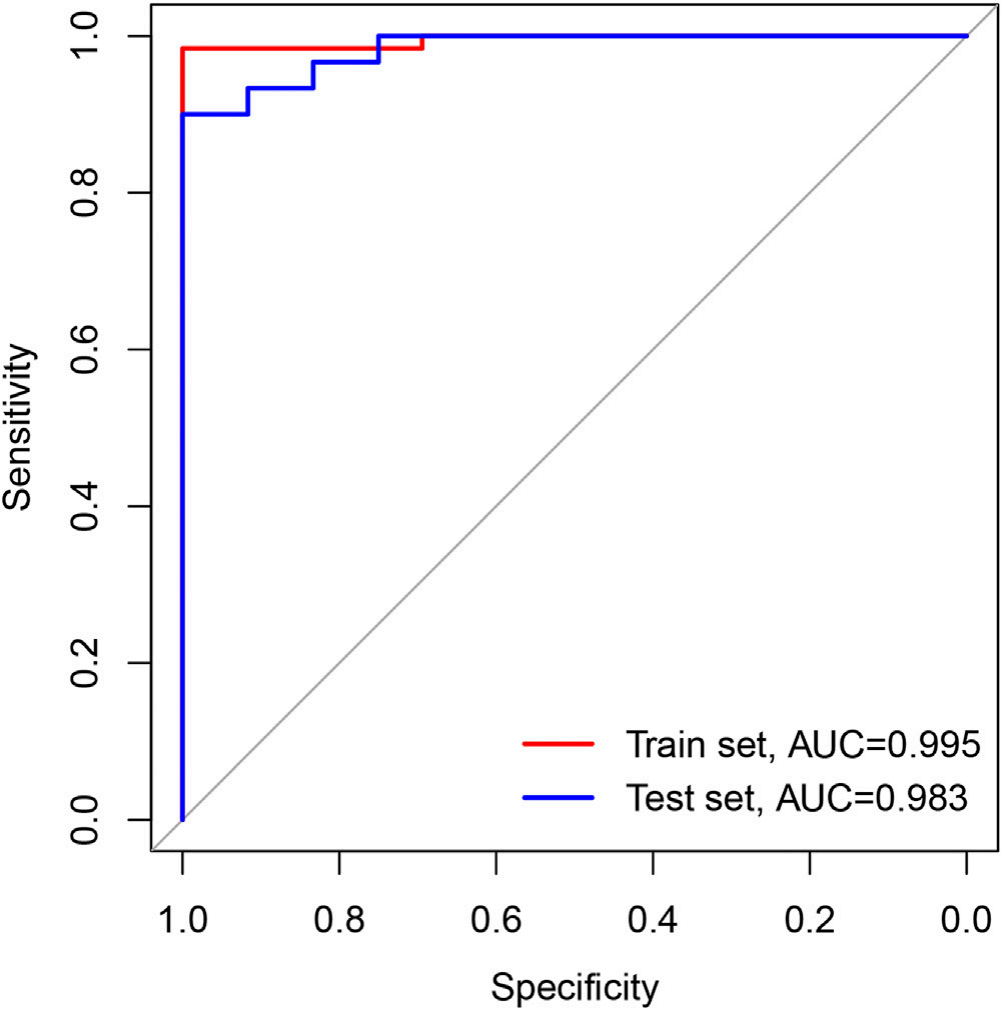
ROC curve based on GEO database. The red line represents train set and the blue line represents test set.

**FIGURE 8:**
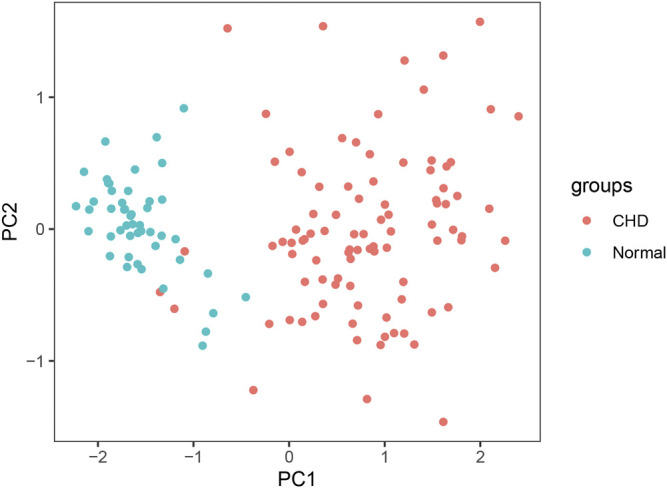
PCA of AC010082.1 and AC011443.1. Red dots represent patients with CHD and light blue dots represent healthy controls.

**FIGURE 9:**
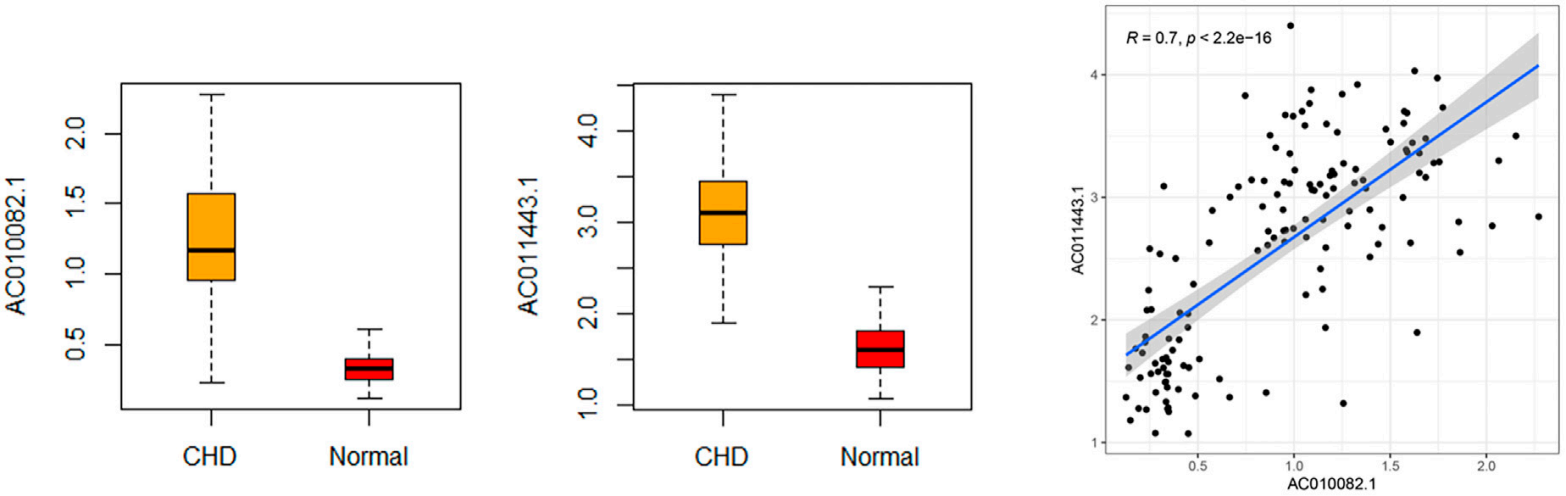
Relative expression level and correlation of AC010082.1 and AC011443.1 based on GEO database.

The sensitivity, specificity, and AUC were 98.41%, 100%, and 0.995, respectively, for the training set, and 93.33%, 91.67%, and 0.983, respectively, for the test set (Figure 7). These two lncRNAs can be regarded as important signatures for the prediction of CHD. PCA of these two lncRNAs can distinguish patients with CHD from healthy controls (Figure 8). At the same time, the results show that AC010082.1 and AC011443.1 are upregulated in patients with CHD (*p* < 0.05), and that AC010082.1 and AC011443.1 have a significant positive correlation (r = 0.7, *p* < 0.01, Figure 9).

**FIGURE 10:**
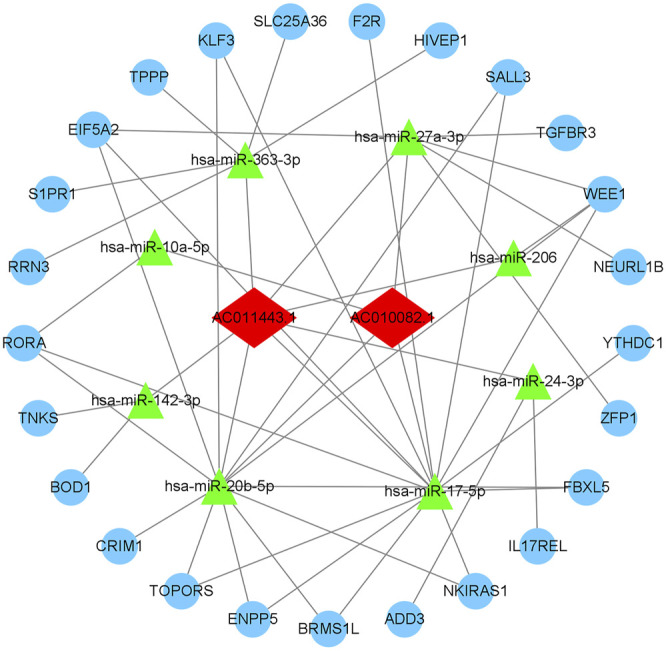
AC010082.1- and AC011443.1-related ceRNA network. The light blue round nodes, red diamond nodes, and green triangle nodes indicate mRNA, lncRNA, and miRNA, respectively.

According to the database targeted prediction analysis, AC010082.1 could bind to hsa-miR-10a-5p, hsa-miR-17-5p, hsa-miR-20b-5p and hsa-miR-27a-3p, and then regulate the expression of 16 mRNAs through MREs. The AC011443.1 could target to hsa-miR-142-3p, hsa-miR-17-5p, hsa-miR-206, hsa-miR-20b-5p, hsa-miR-24-3p, hsa-miR-27a-3p and hsa-miR-363-3p, and regulate the expression of 25 mRNAs (Table 1). These two DElncRNAs could jointly bind to hsa-miR-17-5p, hsa-miR-20b-5p and hsa-miR-27a-3p, thus regulating the expression of 25 mRNAs through MREs and forming the 2lncRNA-8miRNA-25 mRNA -related ceRNA networks (Figure 10).

**TABLE 1:** AC010082.1- and AC011443.1-related target miRNA and target mRNA.

lncRNA	miRNA	mRNA
AC010082.1	hsa-miR-10a-5p	RORA
AC011443.1	hsa-miR-142-3p	BOD1/TNKS
	hsa-miR-206	WEE1
	hsa-miR-24-3p	ADD3/IL17REL
	hsa-miR-363-3p	HIVEP1/RRN3/S1PR1/SLC25A36/TPPP
AC010082.1 and AC011443.1	hsa-miR-17-5p	BRMS1L/EIF5A2/ENPP5/F2R/FBXL5/KLF3/NKIRAS1/RORA/SALL3/TOPORS/WEE1/YTHDC1
	hsa-miR-20b-5p	BRMS1L/CRIM1/EIF5A2/ENPP5/FBXL5/KLF3/NKIRAS1/RORA/SALL3/TOPORS/WEE1
	hsa-miR-27a-3p	EIF5A2/NEURL1B/TGFBR3/WEE1/ZFP1

### External Validation of AC010082.1 and AC011443.1 Using RT-qPCR

**FIGURE 11:**
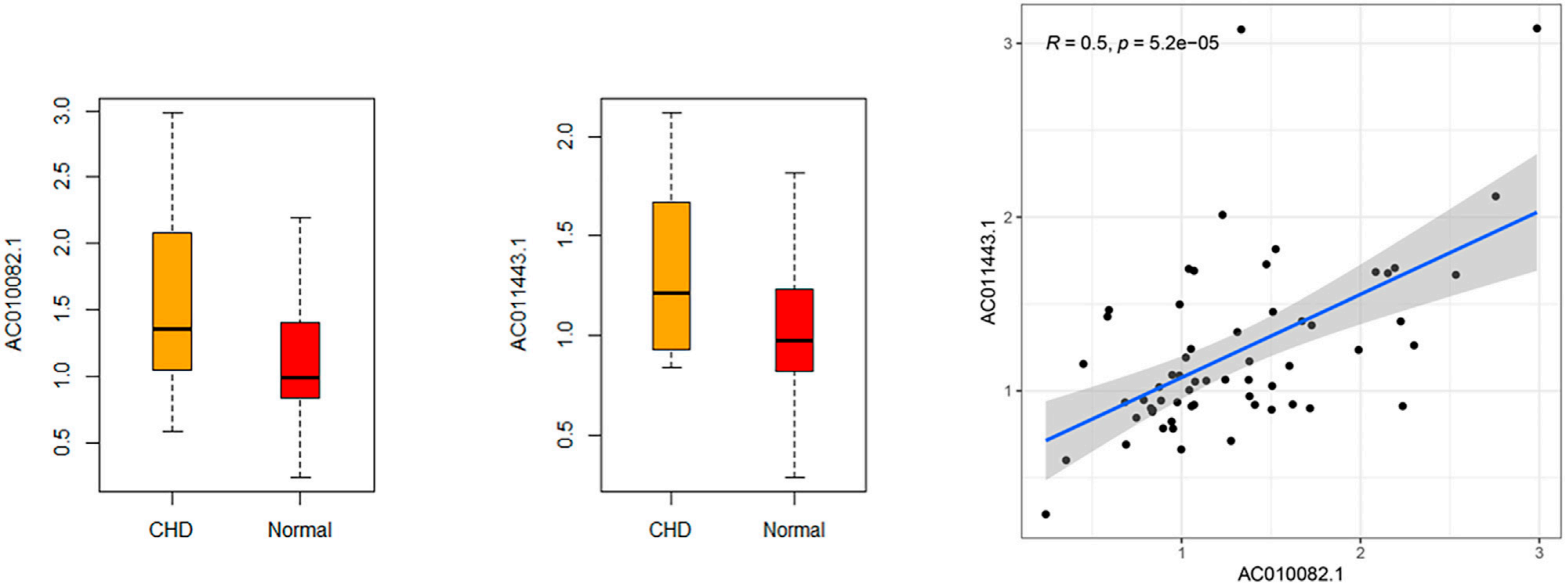
Relative expression level and correlation of AC010082.1 and AC011443.1 in the validation cohort.

**FIGURE 12:**
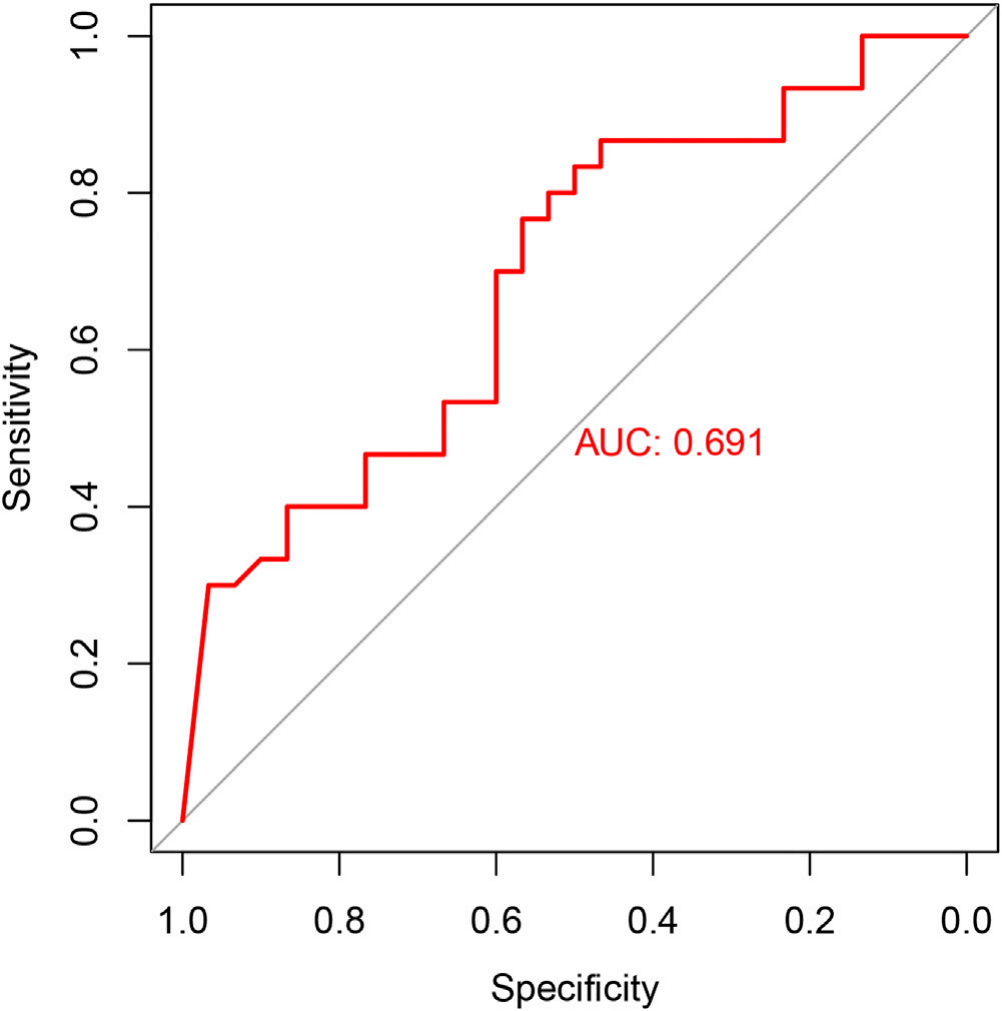
ROC curve in the validation cohort.

RT-qPCR was used to verify the relative expression of AC010082.1 and AC011443.1 in the validation cohort. The results show that AC010082.1 (*p* = 0.009 < 0.01) and AC011443.1 (*p* = 0.02 < 0.05) were significantly higher in the CHD group than in the healthy control group, and the AC010082.1 and AC011443.1 expression levels were significantly positively correlated (r = 0.5, *p* < 0.01, Figure11). Simultaneously, the relative expression levels of AC010082.1 and AC011443.1 were inserted in the prediction model formula Logit(P) = −13.718 + AC010082.1*8.099 + AC011443.1*3.825. The sensitivity, specificity, and AUC of the prediction model were 63.3%, 60.0%, and 0.691, respectively, implying that the model can effectively identify CHD (Figure 12).

## Discussion

Despite considerable advances in modern medicine, CHD diagnosis remains difficult, particularly in the early stages. LncRNA is an important regulator of gene expression, and one of its regulatory mechanisms is ceRNA. MiRNAs can bind to mRNA through MREs, resulting in mRNA degradation or translation inhibition. When lncRNAs and mRNAs have the same MREs, lncRNAs can compete with mRNA for binding to miRNA to prevent mRNA degradation and achieve indirect regulation of mRNA expression level (Wang et al., 2021). The ceRNA mechanism provides a new method for studying the biological functions of lncRNAs and has garnered extensive attention to date (Li et al., 2021b; Lin et al., 2021; Ma et al., 2021).

In this study, a ceRNA network consisting of 38 lncRNAs, 21 miRNAs, and 40 mRNAs was constructed using bioinformatics analysis. KEGG function enrichment in this network was mainly concentrated in the TGF-*β* signaling pathway, TNF signaling pathway, and leukocyte transendothelial migration. These signaling pathways have been previously shown to be closely related to the pathological mechanisms of CHD (Shen et al., 2020; Guo et al., 2021; Sluiter et al., 2021). The TGF-*β* signaling pathway can regulate the proliferation and migration of vascular smooth muscle and endothelial cells, and affect the stability of plaque, which plays an important role in CHD pathogenesis (Zeng et al., 2016). The TNF signaling pathway is involved in myocardial I/R injury and cardiomyocyte apoptosis (Tan et al., 2018). Leukocyte transmigration is critical to the inflammatory response and accelerates the progression of atherosclerosis (Muller, 2011; Bhui and Hayenga, 2017). These DElncRNAs can interfere with pathological changes in CHD through various signaling pathways.

However, further lncRNA analysis using the logistic stepwise regression prediction model revealed that AC010082.1 and AC011443.1 might be important biomarkers of CHD, which was confirmed using RT-qPCR. AC010082.1 is an antisense molecule with a length of 546 bp and is located on chr7:18,429,062–18,430,738, whereas AC011443.1 is also an antisense molecule with a length of 365 bp and is located on chr19:39,134,882–39,136,463. Unfortunately, no relevant studies have been conducted on these two lncRNAs, and their direct mechanism of action remains unclear. Through bioinformatics analysis, it was found that these two DElncRNAs affect the expression of 25 mRNAs by regulating eight miRNAs. The hsa-miR-17-5p, hsa-miR-20b-5p and hsa-miR-27a-3p were jointly regulated by these two DElncRNAs, which may contain a common MRE and have a regulatory relationship through ceRNA mechanism. We found that AC010082.1 and AC011443.1 were overexpressed in patients with CHD and showed a significant positive correlation. The mechanism may be that when lncRNA AC010082.1 is highly expressed in patients with CHD, it will bind more miRNAs, so as to release the inhibition of miRNA on the other lncRNA AC011443.1, thus leading to its high expression (Salmena et al., 2011). AC010082.1 and AC011443.1 bind to miRNA through MREs, thus affecting mRNA expression and biological functions.

Interestingly, we found that hsa-miR-17-5p, hsa-miR-20b-5p and hsa-miR-27a-3p co-regulated by these two DElncRNAs have important biological roles in CHD. The expression of hsa-miR-17-5p is down-regulation in patients with CHD, which could be as a biomarker of CHD (Zhelankin et al., 2021) and a good predictor of the severity of coronary atherosclerosis (Chen et al., 2015). In addition, lncRNA SNHG16 could regulate NF-*κ*B signaling pathway by binding hsa-miR-17-5p, and then affect proliferation and inflammatory response in atherosclerosis patients, which provide a potential target for treating AS (An et al., 2019). Furthermore, miR-20b-5p plays an important role in mediating cardiomyocytes apoptosis via the HIF-1 α/NF- *κ* B pathway (Zhou et al., 2020). And circHIPK3 acting as miR-20b-5p sponge could accelerate cardiomyocyte autophagy and apoptosis during myocardial I/R injury (Qiu et al., 2021). Reportedly, miR-27a expression is upregulated in patients with CHD, and its expression level is remarkable correlated with the severity of coronary artery stenosis, which can be used as a biomarker of CHD (Babaee et al., 2020). Meanwhile, miR-27a may lead to the progression of atherosclerotic plaques by downregulating *ABCA1* and *ABCG1* gene expression (Rafiei et al., 2021).

Besides, these two DElncRNAs affected TGFBR3 expression by co-regulating the miR-27a-3p sponge via bioinformatics prediction. TGFBR3 expression is significantly upregulated in patients after myocardial infarction and may serve as a therapeutic target and biomarker for myocardial damage by activating p38 MAPK to induce cardiomyocyte apoptosis (Chu et al., 2011; Chen et al., 2019). Moreover, it has been found that lncRNA SOX2-OT exacerbates hypoxia-induced cardiomyocyte injury by regulating the miR-27a-3p/TGFβR1 axis (Yang and Lin, 2020). Therefore, we speculated that AC010082.1 and AC011443.1 play important roles in the pathological progression of CHD through the ceRNA mechanism and are potential biomarkers of CHD.

The present study had some limitations. First, we only verified the possibility of using AC010082.1 and AC011443.1 as biomarkers through RT-qPCR, and the clinical samples were obtained from only one research center. In future, larger sample sizes and data from multiple centers countrywide are required to verify our findings. In addition, *in vitro* and *in vivo* experiments to further explore the functions of AC010082.1 and AC011443.1, and the related pathological mechanisms, are warranted.

In summary, our study identified AC010082.1 and AC011443.1 as possible biomarkers of CHD through bioinformatics analysis and RT-qPCR. The ceRNA network associated with these lncRNAs possibly plays a key role in the pathological process of CHD.

## Data Availability

The datasets presented in this study can be found in online repositories. The names of the repository/repositories and accession number(s) can be found in the article/Supplementary Material.
